# Modulation of the von Willebrand factor-dependent platelet adhesion through alternative proteolytic pathways

**DOI:** 10.1016/j.thromres.2011.11.021

**Published:** 2012-04

**Authors:** Nikolett Wohner, András Kovács, Raymund Machovich, Krasimir Kolev

**Affiliations:** Department of Medical Biochemistry, Semmelweis University, Budapest, Hungary, 1094 Tűzoltó u. 37–47

**Keywords:** von Willebrand factor, plasmin, thrombin, neutrophil elastase, matrix metalloproteases, platelets

## Abstract

**Introduction:**

Platelet adhesion to collagen under high shear rates depends on the optimal size of the von Willebrand factor (VWF) multimers, which is determined by their limited proteolysis. The present study attempts to identify the role of hemostatic-fibrinolytic enzymes (thrombin, plasmin) and leukocyte-derived proteases (matrix metalloproteinase (MMP)-8, MMP-9, neutrophil elastase) in the cleavage of VWF and to characterize the effect of flow and platelets on this proteolysis and its functional consequences on platelet adhesion.

Methods and results

According to VWF immunoblots, plasmin, neutrophil elastase and thrombin at concentrations of *in vivo* relevance resulted in extensive degradation of VWF within several minutes. Platelets protected VWF against this proteolysis under static conditions, whereas perfusion of the proteases at 3350 s^-1^ shear rate over VWF immobilized on artery cross sections enhanced its degradation and blocked the protective effect of platelets. In parallel with VWF digestion, the examined proteases impaired the VWF-dependent platelet adhesion as reflected in the decreased surface-bound GpIIb/IIIa immunoreactivity following perfusion of collagen-coated surfaces or artery sections with blood and plasmin, neutrophil elastase or thrombin. Within the time frame of minutes no VWF cleavage could be detected under static or flow conditions after exposure to MMP-8 and MMP-9 at concentrations relevant to physiological neutrophil counts.

**Conclusion:**

Our results indicate a shear- and platelet-dependent role for several proteases in the local modulation of the VWF function.

## Introduction

Von Willebrand factor (VWF) is a glycoprotein that can be found in blood plasma, in the α-granules of platelets and in the subendothelial matrix of blood vessels following release from endothelial cells. The main functions of this glycoprotein are essential for normal hemostasis: it promotes platelet adhesion to thrombogenic surfaces through the platelet receptor GpIb under high shear rate conditions and carries factor VIII in plasma prolonging its lifetime in circulation. VWF binds to collagen types I, III, VI and to platelet surface glycoproteins GpIb and GpIIb/IIIa (reviewed in [1]). Binding of VWF to fibrin influences platelet adhesion at the site of vascular injury [2]. The binding of soluble VWF to nonactivated platelets is tightly regulated to prevent aggregation in the circulation, but VWF immobilized onto a surface is highly reactive toward flowing platelets. Exposure to shear rates in the range of 500 and 5000 s^-1^, which is typical for the small arterioles [3] or stenotic coronary arteries [4], appears to be an essential factor in the control of VWF reactivity. While circulating VWF multimers are in coiled conformation that shields the A1 domain from interacting with platelets, binding to a substrate under shear stress extends the shape of the molecule with consequent increase in platelet adhesiveness [5]. Another regulatory tool in the VWF – platelet interactions is the modification of the size of VWF multimers through proteolysis, which decreases the reactivity of VWF. The smallest form of VWF is a dimer of identical disulfide-linked subunits [6]. In the largest multimers the number of subunits can be as many as 50 to 100 while the molecular mass ranges from 540 kDa for the dimer to several thousand kDa for the largest multimers. Proteolytic cleavage by a metalloproteinase (ADAMTS-13) is responsible for reducing the size of large VWF multimers in the plasma by cleaving the subunit at the bond between Tyr842 and Met843 [7]. Proteolysis restricts the positive effects of flow on VWF reactivity, because under shear stress VWF changes its three-dimensional structure [5] which enables the cleavage by ADAMTS-13. Deficiency in the ADAMTS-13 function results in thrombotic thrombocytopenic purpura (TTP) [8,9] but the ADAMTS-13 levels do not always correlate with the severity of TTP [8,10]. Accordingly additional regulatory mechanisms should be considered. *In vivo* adhered platelets recruit leukocytes from circulating blood, predominantly neutrophils, representing 76% of the leukocytes in thrombi [11] that become activated and secrete elastase, cathepsin G and matrix metalloproteinases. The cleavage of VWF by neutrophil granulocyte-derived proteases has been documented [12–15] and the cleavage sites in VWF have been recently identified [16], but no investigations have been carried out under flow conditions or in the presence of platelets and thrombogenic surfaces. The importance of leukocyte-derived serine proteases in the cleavage of oxidized VWF has been recently pointed out as a potential compensatory mechanism for the partial inactivation of ADAMTS-13 by reactive oxygen species originating from the same cells [17].

In thrombi VWF is exposed to additional proteases. Thrombin is generated in the blood coagulation cascade, while plasmin, as a main fibrinolytic enzyme is produced by plasminogen activators from plasminogen. The concentration of these enzymes is in the range above 10 nM in thrombi and this environment also provides a partial protection from plasma inhibitors [18]. About 10 nM thrombin is present in whole blood at the point of clotting [19] and 20 nM neutrophil elastase concentration is expected in cell suspension of degranulated PMNs [20,21]. Data on VWF degradation by plasmin and thrombin are limited [22–24] and the impact of flow and platelets on the proteolytic susceptibility of VWF to these proteases has not been characterized in conjunction with its functional consequences on platelet adhesion to the arterial wall. The present study addresses these aspects of the VWF proteolysis.

## Materials and Methods

### Purified enzymes

Active enzyme concentration of human neutrophil elastase (Serva Electrophoresis Gmbh, Heidelberg, Germany) was determined as described previously [18]. Recombinant human MMP-8 and MMP-9 proenzymes (R&D Systems, Abingdon, England) were activated with 1 mM p-aminophenylmercuric acetate (APMA, Sigma-Aldrich Kft., Budapest, Hungary) at 37 °C for 1 h and 24 h, respectively, and their activity was determined by gelatin substrate zymography [25]. Bovine thrombin was purchased from Serva Electrophoresis Gmbh, Heidelberg, Germany, thrombin was further purified by ion-exchange chromatography on sulfopropyl-Sephadex yielding a preparation with specific activity of 2100 IU/mg [26] and 1 IU/ml was considered equivalent to approximately 10.7 nM by active site titration [27]. MMP2/MMP-9 Inhibitor I [(2R)-2-[(4-biphenylylsulfonyl)amino]-3-phenylpropionic acid] for the inhibition of MMP-9 and MMP-Inhibitor I (4-Abz-Gly-Pro-D-Leu-D-Ala-NHOH) for the inhibition of MMP-8 (Calbiochem, LaJolla, CA) were applied at 5 μM concentration each. Pefabloc (aminoethyl-benzenesulfonylfluoride, Serva Electrophoresis Gmbh, Heidelberg, Germany) was used for the inhibition of serine proteases at a concentration of 10 mM.

### Preparation of human artery cryosections and collagen-coated surfaces

The study protocol was approved by the institutional and regional ethical board. Human iliac artery was removed from deceased healthy organ donors, immediately frozen in 2-methylbutane in dry ice and stored at − 70 °C. Cryosections (6 μm thickness) of the artery were placed on poly-L-lysine-coated slides (“Poly-Prep Slides”, Sigma-Aldrich Kft., Budapest, Hungary) 1–3 days before the experiments and stored at − 20 °C until use. Collagen (Helena Biosciences Europe) was diluted to 100 μg/ml at 4 °C in 1.5 mM KH_2_PO_4_, 8.1 mM Na_2_HPO_4_ buffer pH 7.4 containing 137 mM NaCl and 2.7 mM KCl (PBS), incubated for 15 minutes at 37 °C and 50 μl was applied to poly-L-lysine-coated slides. Polymerisation of collagen took place at 37 °C for 24 hours.

### Proteolysis of VWF under static conditions

In the absence of platelets purified multimeric human VWF (Haemate P500, Helena Biosciences Europe) at 10 μg/ml in 10 mM Hepes 150 mM NaCL pH 7.4 was incubated with the studied enzymes at various concentrations and samples were taken at different times for immunoblotting. In order to prevent the activation of platelets by the proteases, lyophilized platelets (Helena Biosciences Europe) were used when the effect of platelets was evaluated under static conditions and accordingly this assay reflects only properties of platelets independent of activation. In terms of VWF interactions these lyophilized platelets retain their basic hemostatic properties as evidenced by their widely accepted application in clinical laboratories (reviewed in [28]). These experiments were performed in the cuvette of a Carat TX4 four-channel optical aggregometer (Carat Diagnostics, Budapest, Hungary), where gentle stirring maintained the homogeneity of the platelet suspension, but did not change the compact (“static”) conformation of VWF. In order to remove any traces of plasma protease inhibitors lyophilized platelets were washed in 50 mM Tris–HCl 150 mM NaCl pH 7.4 four times and centrifuged for 10 min at 80,000 *g*. Thereafter platelets were suspended in 10 μg/ml VWF solution at 200,000 /μl count and the examined proteases were added at various concentrations. Platelet agglutination was initiated with 1.5 mg/ml ristocetin (Helena Biosciences Europe) and monitored in the aggregometer. In parallel samples were taken for VWF immunoblotting. In some experiments before the aggregometric assay washed lyophilized platelets were preincubated with 50 nM plasmin for 15 minutes followed by inhibition of plasmin with Pefabloc at 10 mM in order to differentiate the functional consequences of cell surface receptor and VWF degradation by the enzyme. In order to check the non-specific absorption of enzymes to platelets, after the wash procedure platelets were resuspended in plasmin solution and following centrifugation as described above plasmin activity (expressed in ΔA_405_/min) in the supernatant was measured on 0.2 mM Spectrozyme-PL (H-D-norleucyl-hexahydrotyrosyl-lysine-*p*-nitroanilide, American Diagnostica, Pfungstadt, Germany).

### Proteolysis of VWF under flow conditions

Artery cross sections on poly-L-Lys-coated or collagen-coated slides were perfused as previously described [29] in a parallel-plate chamber with VWF and lyophilized platelets or with VWF alone. The perfusion chamber was constructed on the slides using a 0.3 cm wide and 1.5 cm long flow channel cut into a piece of double-sided tape (Scotch, 1.27 cm wide, 64 μm thick), which was sandwiched to a methacrylate cover (2.3 cm x 3.8 cm) containing the inlet and outlet tubing (1-mm internal diameter Tygon). Assuming laminar flow conditions, the shear rate at the surface of the section was 3350 s^-1^, according to the formula 1.03*6 *Q*/(w*h^*2*^), where *Q* is the flow rate in ml/s, *w* and *h* are the width and the height of the flow path in cm, respectively [30]. The initial VWF perfusion was followed by a 1.5-min wash with PBS and a 1.5-min perfusion with the examined enzyme solution. The out-flowing solution was collected and the inhibitor of the respective enzyme was added immediately. This solution was concentrated with polyethylene glycol 20.000 (Sigma-Aldrich Kft., Budapest, Hungary) and was used for Western Blot analysis of VWF fragments.

### Western Blot Analysis of VWF and its proteolytic fragments

Load buffer containing 100 mM Tris–HCl 100 mM NaCl, 5%(w/v) SDS, 5%(v/v) β-mercapto-ethanol, pH 8.2 was added to the samples collected in the proteolytic reactions at a ratio of 1:4 followed by heat-treatment at 95 °C for 3 min. After gel electrophoresis, samples were electroblotted onto nitrocellulose membranes, and VWF along with its degradation products were detected by polyclonal rabbit anti-human VWF immunoglobulin G (Sigma-Aldrich Kft., Budapest, Hungary) followed by goat-anti-rabbit IgG peroxidase conjugate. The captured secondary antibodies were visualized using the ECL Western blotting analysis system (GE Healthcare, Uppsala, Sweden).

### Visualization of adherent platelets

Indirect immunofluorescence microscopy was used to detect platelets adhered to the tissue samples. The sections were blocked with 200 μl 50 mM Tris–HCl, 100 mM NaCl, 0.02%(w/v) NaN_3_ pH 7.4 (TBS) containing 20 g/l bovine serum albumin (BSA) for 30 min followed by washing with TBS. Afterwards they were incubated for 60 min with 100 μl 4 μg/ml mouse monoclonal antibody against human CD41 GpIIb/IIIa (Biodesign International, Saco, USA) diluted in TBS-BSA. The slides were washed three times with TBS and 100 μl 2 μg/ml Alexa Fluor 488 goat-anti-mouse IgG (Invitrogen, Budapest, Hungary) diluted in TBS-BSA was added and incubated for 30 min. The slides were washed 3 times and glass coverslips were affixed over a drop of 50%(v/v) glycerol in TBS. Confocal fluorescent images were taken from the slides using a Zeiss LSM510 confocal laser scanning microscope equipped with a 20 × 1.4 objective (Carl Zeiss, Jena, Germany). The green fluorescent signal was acquired using a 488-nm excitation laser line (10% intensity) and emission was detected in the 500–530 nm wavelength range. All steps were carried out at 22 °C, at which temperature no proteolytic degradation of platelet receptors by plasmin can be detected [31].

### Data analysis of platelet adhesion to the vessel wall

The quantification of platelets adhered to the vessel wall was performed with the Scion Image software (Scion Corp, Frederick, Maryland) selecting the region of interest, calculating its surface area in pixels and setting a threshold intensity value for automatic identification of the area covered by adhered platelets. The area covered by platelets is reported in percentage of the whole area as mean ± S.E.M. from *n =* 5 images. For estimating the statistical significance of differences Student's two-sample *t*-test was used.

## Results

### Proteolysis under static conditions in the absence of platelets

**Fig. 1:**
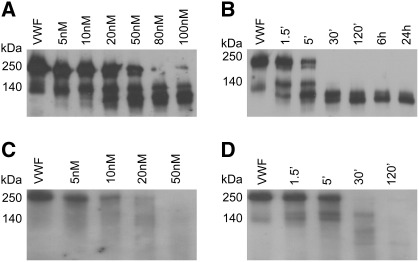
**Degradation of VWF by plasmin and neutrophil elastase under static conditions.** Purified human VWF (10 μg/ml) was incubated for 30 min with plasmin (A) or neutrophil elastase (C) at the indicated concentrations or for various times with 100 nM plasmin (B) or 50 nM neutrophil elastase (D) at 37 °C. Proteolytic cleavage products were detected by Western Blot analysis as described in Materials and Methods.

The degradation of VWF was evaluated at protease concentrations with physiological relevance. Plasmin at 5 nM, a concentration that is sufficient to completely dissolve fibrin (its primary intravascular substrate) within 30 min [32] generated VWF degradation products at the lowest limit of detectability within the same time interval (Fig. 1A). At concentrations relevant to thrombolytic therapy (over 100 nM) [33] plasmin digested the intact VWF monomers almost completely at 30 min and initial degradation products could be detected as early as after 1.5 min incubation (Fig. 1B). Although neutrophil elastase generated different proteolytic products (Fig. 1C), its efficiency in degrading VWF appears to be similar to that of plasmin based on the time course of digestion with comparable enzyme concentrations (Fig. 1D). In contrast to plasmin and elastase, MMP-9 and MMP-8 at 20 nM, a concentration expected in suspensions of neutrophil leukocytes with physiological cell count [34] degraded VWF very slowly and cleavage products could be detected only after 24-h digestion. The efficiency of thrombin in VWF degradation was similarly low. No cleavage products could be detected after 30 min digestion even at 100 nM thrombin, a concentration 10-fold higher than the values observed in blood at the point of clotting [19] (not shown). The presence of 1 mM CaCl_2_ did not affect the time course and the enzyme concentration dependence of VWF degradation by plasmin and elastase evaluated as described for Fig. 1 (data not shown) and accordingly calcium effects were not investigated further in different experimental setups.

### Proteolysis under static conditions in the presence of platelets

**Fig. 2:**
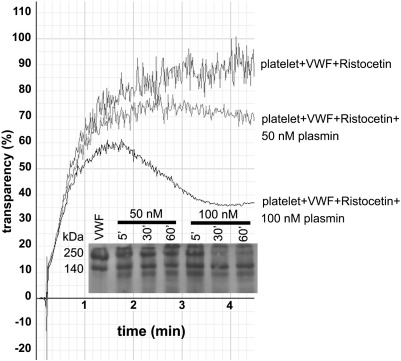
**VWF proteolysis under static conditions in the presence of platelets.** Ristocetin (1.5 mg/ml) was used to initiate agglutination of 200,000 /μl platelets in 10 μg/ml VWF solution and the transparency of the suspension was monitored in an aggregometer. Plasmin was added to the platelet suspension before ristocetin where indicated. Representative aggregation curves of 5 measurements are shown. The inset shows a parallel immunoblot of the same sample for the detection of VWF cleavage products.

The effects of platelets on the proteolysis of VWF were evaluated in two assay systems: ristocetin-induced platelet agglutination to monitor the changes of VWF function and immunoblot to detect intact and fragmented VWF monomers (Fig. 2). Plasmin decreased the maximal aggregation of platelets and later the aggregates disassembled (Fig. 2), but this effect was not observed if platelets were pre-treated with plasmin and the enzyme was inhibited prior the agglutination assay suggesting that this loss of function is based on VWF and not surface receptor degradation. Such a conclusion is supported by the parallel immunoblots of the same samples, which indicated VWF cleavage products (Fig. 2, Inset), but the time course of their appearance was significantly slower compared to the proteolytic rate in the absence of platelets (Fig. 2, Inset vs. Fig. 1B). The rest of the evaluated proteases (neutrophil elastase, thrombin, MMP-8, MMP-9) did not produce any cleavage products in the presence of platelets and did not lower the maximal aggregation when applied at concentrations with detectable proteolytic effect in the absence of platelets (data not shown). Ristocetin in the absence of platelets did not affect VWF digestion with any of the examined proteases (data not shown). Thus, we can conclude that platelets protect VWF against proteolysis by various proteases probably through decreased lytic susceptibility of the cell-bound VWF. Non-specific absorption of enzymes to platelet surfaces cannot account for this effect, because according to our amidolytic activity assay, following sedimentation of lyophilized platelets suspended in plasmin solution supernatants retain approximately 80% of the original enzymatic activity.

### Proteolysis under flow conditions

**Fig. 3:**
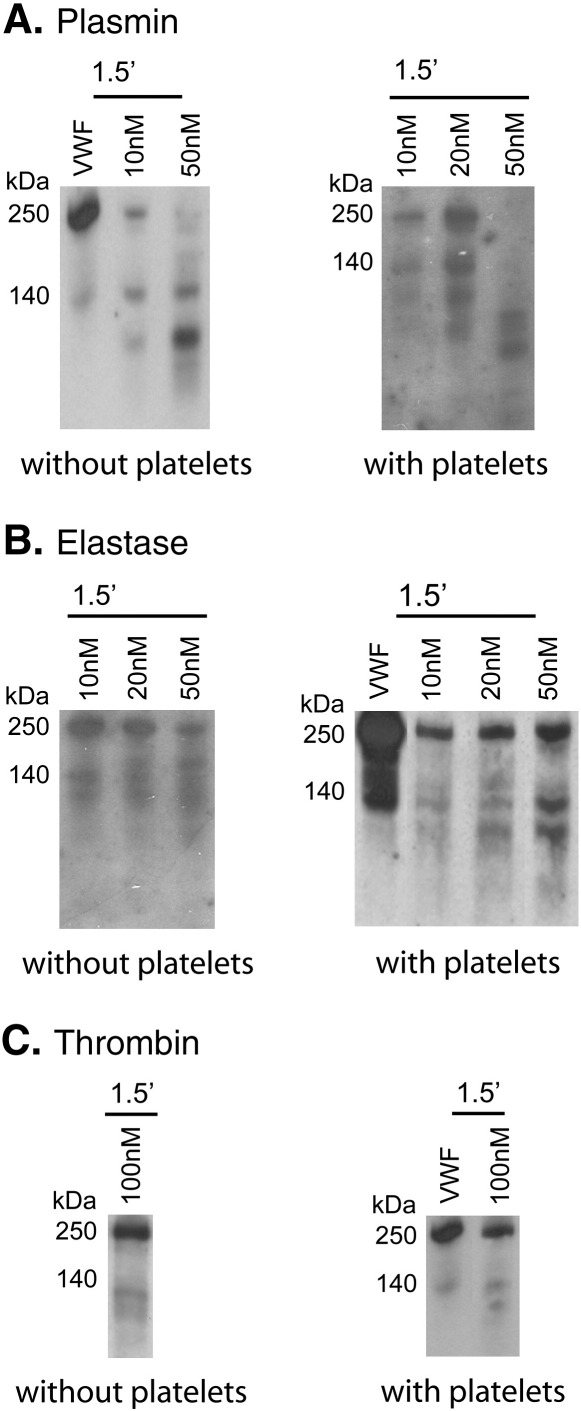
**Cleavage of VWF by plasmin, neutrophil elastase and thrombin under flow conditions.**VWF (10 μg/ml) alone or in a mixture with 200,000/μl lyophilized platelets was perfused at 3350 s^-1^ shear rate over collagen surface as described in Materials and Methods. Thereafter plasmin (A), neutrophil elastase (B) or thrombin (C) at the indicated concentrations was perfused over the surface at the same flow rate. VWF cleavage products in the outflowing solution were detected by Western Blot analysis.

Proteolysis of VWF was evaluated in a flow chamber, where VWF was immobilized to an artery cross-section followed by protease perfusion at 3350 s^-1^ shear rate on the surface (Fig. 3). Shear forces appear to sensitize VWF to the action of proteases. The 1.5-min perfusion resulted in a more extensive degradation by plasmin compared to the static digestion with identical enzyme concentrations (Fig. 3A vs. Fig. 1). Proteolysis by neutrophil elastase and thrombin was affected to a lesser degree by shear, whereas MMP-8 and MMP-9 at the abovementioned physiologically relevant concentration (20 nM) did not cleave VWF even under the applied flow conditions (data not shown). In contrast to the static system, in the perfusion chamber platelets did not protect VWF against proteolysis by neutrophil elastase and thrombin (Fig. 3B and C), but importantly, enhanced the degradation by plasmin as indicated by the increase in the fraction of fragments with size smaller than 140 kDa in Fig. 3A.

### Modulation of VWF function by proteolysis under flow conditions

**Fig. 4:**
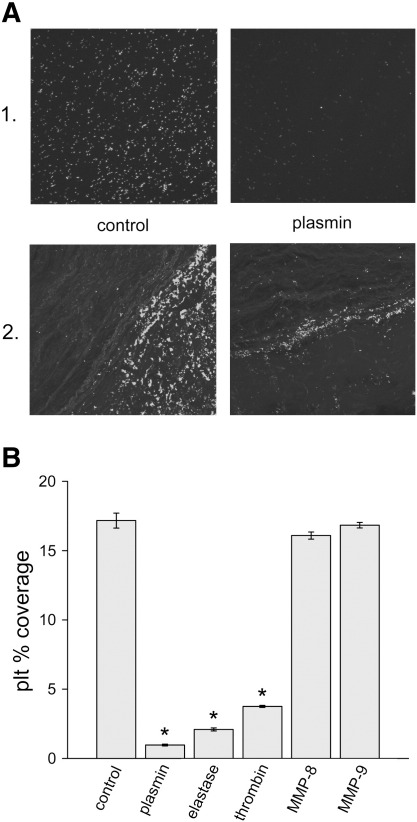
**Platelet coverage of thrombogenic surfaces.** Collagen surface (1.) or arterial cryosection (2.) was perfused with citrated whole blood for 1.5 min at 3350 s^-1^ shear rate in the flow chamber described in Materials and Methods followed by a perfusion of PBS (control) or 10 nM plasmin. Platelets were visualized by indirect immunofluorescence (A) and images were analyzed for platelet coverage before (control) and after the perfusion with plasmin (10 nM), neutrophil elastase (10 nM), thrombin (100 nM), MMP-8 (20 nM), MMP-9 (20 nM). In panel B the area of collagen surfaces covered by platelets is shown as percentage of the total area (mean ± SEM, *n* = 5, * p < 0.01).

VWF-dependent platelet adhesion was evaluated on artificial collagen surfaces or artery cryosections. Following initial perfusion with blood, platelets adhered to the surface and could not be detached by perfusion of enzyme-free buffer at 3350 s^-1^ shear rate (Fig. 4A). Plasmin, neutrophil elastase and thrombin perfusion significantly decreased platelet adhesion while matrix metalloproteinases had no effect (Fig. 4B).

## Discussion

The data presented in this report show that proteases with well-known function in the resolution of blood clots (plasmin, neutrophil elastase) extensively degrade VWF (Fig. 1) at concentrations with efficient fibrinolytic activity [32] and thus extend the concept of transient compartments in hemostatic or pathological thrombi [35] with a new mechanism. The disassembly of the clots may be attributed not only to the dissolution of the fibrin network, but also to the break-down of the adhesive molecular links between platelets and blood vessel wall formed by VWF multimers. This suggested role is supported by our results from a flow model of platelet adhesion to artery cross sections and collagen, in which the VWF dependence of adhesion has been previously characterized [36]. Here the same proteases detach platelets adhered to collagen or artery wall sections at concentrations that efficiently degrade VWF (Fig. 4). Importantly, not all neutrophil proteases appear to be involved in this mechanism. Here we could not detect cleavage of VWF by MMP-8 and MMP-9 at 20 nM, a concentration that we have recently shown to be efficient in the proteolytic modification of the arterial wall structure [34]. This result is in agreement with a recent report [16] demonstrating no VWF degradation by MMP-9 under static conditions and partial digestion only under shear and at higher enzyme concentrations (50 nM or more). Thus, MMP-8 and MMP-9 appear to favour platelet adhesion rendering the arterial wall more thrombogenic [34] and sparing VWF (present results) in contrast to elastase, which increases the adhesiveness of the vessel wall [34], but at a later stage promotes platelet detachment (Fig. 4) through VWF degradation (Figs. 1C&D and 3).

At sites of vascular injury ultra-large VWF multimers (of more than 40 monomeric subunits) are released from their storage granules in endothelial cells [37] and platelets [38]. Their hyperactivity [39] is essential for shear-induced platelet aggregation and rapid formation of hemostatic plugs. However, the propagation of such hyperactive VWF derivatives in the systemic circulation would cause a prothrombotic state as observed in TTP. This detrimental situation is normally prevented via VWF cleavage by ADAMTS-13 on the surface of endothelial cells [40] or platelets [41]. Here we demonstrate several alternative possibilities for negative feed-back control of VWF function at sites of platelet adhesion and activation. Platelets support procoagulant reactions (reviewed in [42]), the final outcome of which is fibrinogen-fibrin conversion catalyzed by thrombin. At the point of blood clotting the concentration of thrombin is 10 nM [19] and at this concentration thrombin further supports platelet adhesion through modifications of the arterial wall structure [34], but it does not cleave VWF (present results) and thus the adhesive properties of VWF remain unaltered. At later stages of whole blood clotting, the thrombin concentration rises to values of several hundred nM [19]. Such concentrations of thrombin are sufficient to cleave VWF under flow (Fig. 3) and thus excessive and sustained generation of thrombin would restrict the availability of hyperactive VWF to the locus of its release. At later stages of thrombus development, when the classic (plasminogen-dependent) and alternative (leukocyte-dependent) [43] fibrinolytic pathways are activated, additional proteases with the potential to modify the size of VWF multimers (plasmin, neutrophil elastase) are recruited as discussed above. The local proteolytic potential for VWF cleavage can be highly variable, because the activity of thrombin, plasmin, neutrophil elastase depends on multifactorial activation or cellular release [25], but at the concentrations used in the present study these proteases appear to be even more efficient than ADAMTS-13. According to an earlier report [44] the half-life of VWF is more than 3 h when exposed *in vitro* to 6.7 nM ADAMTS-13 corresponding to its physiological plasma level [45], whereas our data evidence more extensive VWF degradation within 30 min at enzyme concentrations of similar magnitude and physiological relevance (Fig. 1). The direct effects of thrombin and plasmin on VWF may compensate for the known inactivation of ADAMTS-13 by these proteases [46].

Shear forces [5,16] and ristocetin [47] modify the conformation of VWF so that it can bind the GpIb receptor of platelets with consequent adhesion or agglutination. In our proteolytic assay under flow conditions (Fig. 3) the increased partial degradation of VWF by plasmin, neutrophil elastase and thrombin suggests that shear-induced conformational change promotes a more general proteolytic susceptibility similar to that reported for ADAMTS-13 [8]. The presence of platelets further sensitizes VWF to plasmin cleavage under flow (Fig. 3), thus contributing to the fragility of platelet-rich thrombi exposed to high shear stress. According to this result suppression of plasmin activity in the initial stages of hemostatic plug formation appears to be essential in a post-injury state. Thus, the hemorrhagic trend in patients with plasminogen activator inhibitor-1 deficiency can be attributed not only to the failure of the anti-fibrinolytic defense [48], but also to the compromised stability of platelet adhesion. In view of the sensitizing effect of shear on VWF degradation by the reported serine proteases, the flow-borne large mass of plasma protease inhibitors acquires an essential supportive role in the propagation phase of thrombus formation *in vivo* as a factor opposing the local proteolysis and thus protecting the adhesive molecule.

In contrast to proteolysis under flow, platelets block VWF degradation partially (with plasmin, Fig. 2) or completely (with neutrophil elastase and thrombin, not shown) under static conditions. Through the maintenance of VWF integrity, this interplay of platelets, VWF and plasmin appears to act as a synergetic mechanism in the recently described VWF-dependent protection of fibrinogen against plasmin [23]. A concept for self-supporting interplatelet-cohesion circuit emerges from these results: platelets moderate the digestion of VWF, which in turn protects fibrinogen against plasmin [23] and finally both adhesive molecules contribute to the intercellular bridges between platelets [49]. Thus, in compartments devoid of shear effects (occlusive thrombi, vessels with slow blood flow) the stability of blood clots may be largely dependent on the proteolytic protective effects of platelets on all three cohesive molecules (fibrin, fibrinogen and VWF) in thrombi.

The present study evaluated the alternative proteolysis of VWF in association with its functional consequences in shear-dependent platelet adhesion. For the first time we show that plasmin, neutrophil elastase and thrombin can detach platelets anchored at high shear stress in a VWF-dependent manner to collagen matrix or inner layers of arteries (Fig. 4). Because under the conditions of our experiments the platelet receptors (GpIb, GpIIb/IIIa) are known to be protected against proteolysis with plasmin [31], this anti-adhesive effect of the proteases can be attributed to the digestion of VWF, which is clearly demonstrated with the immunoblots of perfusate from the platelet-adhesion assay (Fig. 3). These results concerning platelet adhesion are in agreement with earlier studies on the inhibition of shear stress-induced platelet aggregation by plasmin, which has been shown to be the consequence of degradation of VWF and not of platelet receptors [50]. In our static experimental setup (Fig. 2) only plasmin could reduce ristocetin-induced platelet agglutination in parallel with VWF degradation (Fig. 2, Inset). Proteases (neutrophil elastase, thrombin), which do not digest VWF in the presence of platelets, could not suppress ristocetin-induced platelet agglutination in our assay system. This result supports the abovementioned conclusion that in the shear-dependent assay proteolysis of VWF (and not platelet receptors) is responsible for the detachment of adhered platelets when exposed to plasmin, neutrophil elastase or thrombin.

In conclusion, the present study confirms the potential of proteases with known occurrence in thrombi to modify the platelet adhesive function of VWF under high shear stress and proves that this effect depends on proteolysis exerted at physiologically relevant enzyme concentrations. This alternative route for control of VWF function is probably restricted to local compartments in blood clots, where these proteases are relatively protected against their abundant plasma inhibitors.

## Conflict of interest

None to declare.
